# IgE+ plasmablasts predict the onset of clinical allergy

**DOI:** 10.3389/fimmu.2023.1104609

**Published:** 2023-02-02

**Authors:** Elisabeth M. Simonin, Susanna Babasyan, Justine Tarsillo, Bettina Wagner

**Affiliations:** Department of Population Medicine and Diagnostic Sciences, College of Veterinary Medicine, Cornell University, Ithaca, NY, United States

**Keywords:** allergy, hypersensitivity, IgE (immunoglobulin E), plasmablast, biomarker

## Abstract

**Introduction:**

IgE+ plasmablasts develop following allergen exposure and B cell activation. They secrete IgE and therefore are directly linked to maintain the mechanisms of IgE-mediated allergies. Here, we show that the presence of IgE+ plasmablasts in peripheral blood not only coincides with clinical allergy, but also predicts the upcoming development of clinical disease.

**Methods:**

Using an equine model of naturally occurring allergy, we compared the timing of allergen exposure, arrival of IgE+ plasmablasts in peripheral blood, and onset of clinical disease.

**Results:**

We found that IgE+ plasmablasts predict the development of clinical allergy by at least 3 weeks and can be measured directly by flow cytometry or by IgE secretion following *in vitro* culture. We also compared the IgE secretion by IgE+ plasmablasts with total plasma IgE concentrations and found that while IgE secretion consistently correlates with clinical allergy, total plasma IgE does not.

**Discussion:**

Together, we describe IgE+ plasmablasts as a reliable and sensitive predictive biomarker of allergic disease development.

## 1 Introduction

Immunoglobulin E (IgE) causes allergic reactions through binding to IgE receptors on effector cells and stimulation by specific allergens (1). Allergen-specific IgE is often used to diagnose allergy development and severity (2, 3). Testing for allergen-specific serum IgE can also guide which allergens should be included in allergen immunotherapy (4) and predict treatment effectiveness (3). However, the concentration of allergen-specific IgE in circulation can be low and the identification of causal allergens can be difficult in part due to cross reactivity between major and minor allergens (5) and missing information of which allergen epitopes are clinically relevant (6). In addition, IgE levels may not predict treatment success, for example as was seen in an Omalizumab trial (7). Due to this variability, allergic patients can be surprised by unexpected allergic reactions to new allergens or left unsure of the true disease severity. In addition, there are currently no biomarkers to determine whether allergen immunotherapy treatment is effectively desensitizing the individual. As a result, there is need for an allergy biomarker that can better predict and monitor allergy development and severity and can also guide treatment decisions.

IgE is produced when allergen-specific B cells are activated by allergen in the presence of the cytokines IL-4 and IL-13 (8–10), leading to class switch recombination of the constant region to express the IGHE gene (11, 12). These class-switched B cells, now expressing an IgE B cell receptor, then receive additional survival signals and begin differentiating into memory B cells and plasma cells, which secrete high concentrations of IgE into circulation (13–15). During this activation process, some IgE+ B cells differentiate into IgE+ plasmablasts. IgE+ plasmablasts secrete antibodies and enter peripheral blood, providing a snapshot of the IgE+ B cell to plasma cell differentiation process simultaneously occurring in the lymph node or local tissue. Peripheral IgE+ plasmablasts may home to the bone marrow where they further differentiate into plasma cells (16).

We have recently characterized IgE+ plasmablasts as IgE secreting cells in peripheral blood that positively correlate to both clinical allergy severity and secreted IgE concentrations (17). Due to the close connection between B cell activation by allergen and their differentiation into IgE+ plasmablasts, we proposed that circulating IgE+ plasmablasts could be a direct marker of clinical allergy development and severity. To explore the relationship between allergen exposure, IgE+ plasmablasts in the periphery, and the development of clinical disease, we used a natural horse allergy model of *Culicoides (Cul)* hypersensitivity (18). Horses can naturally develop *Culicoides (Cul)* hypersensitivity, which is an IgE-mediated allergic response (19) to salivary proteins of *Cul* midges (20–28). When exposed to *Cul* midges in the environment, allergic horses undergo an immediate hypersensitivity reaction and develop clinical signs including pruritis, dermatitis and alopecia at bite sites, which can persist for several months in the summer (25, 29).

*Cul* hypersensitivity is seasonal and recurrent. All individuals in our study, allergic and healthy, lived together and were exposed to the same environment and frequency of allergen exposure, through *Cul* bites, in the summer. Allergic horses, therefore, were exposed to allergen and developed clinical allergy in synchrony (30). Typically, signs of clinical allergy developed 3-4 weeks after allergen exposure began, providing an initial window of time when horses were exposed to allergen but did not yet show clinical disease (17). We used this allergy model to ask what the relationship is between allergen exposure and the migration of IgE+ plasmablasts into peripheral blood, and if IgE+ plasmablasts precede clinical allergy.

## 2 Materials and methods

### 2.1 Animals, sample collection, and allergy scoring

All experiments were conducted on samples from Icelandic horses with and without clinical allergy (**Table 1**). All horses lived together in the same environment with similar natural exposure to *Cul* midges from mid-April to mid-October. *Cul* were absent from the environment during winter months. Vaccination and deworming were synchronized. All horses were annually vaccinated against rabies, tetanus, West Nile virus and Eastern and Western Encephalitis virus, as well as dewormed with moxidectin and praziquantel (Zoetis, Parsipanny, NJ, USA) once a year in December. All horses were on the same diet. They were kept full time on large pastures with run-in-sheds, free access to water, mineral salt blocks, were grazing in the summer and fed grass hay in the winter.

**Table 1:** Horses and clinical signs of allergy.

Allergy status^a^	Sex (count)	Age Median (range)	Preclinical Phase Clinical score Median (range)^b,c^	Allergic Phase Clinical Score Median (range)^b,d^	Resolving Phase Clinical Score Median (range)^b,e^
Healthy	Mare (5) Gelding (5)	8 (8-10) 9 (8-10)	0 (0) 0 (0-1)	0 (0-1) 0 (0-0.5)	0 (0-2.5) 0 (0-2.5)
Severe Allergy	Mare (n=5) Gelding (n=1) Stallion (n=1)	17 (12-18) 10 9	1 (0-2.5) 1 (0-1.5) 2 (0-3.5)	6.5 (1-10) 3 (1-7) 6.5 (2.5-9)	3.8 (1-9.5) 3 (1-4.5) 1.5 (1.5-2)

a. Allergic horses are all confirmed to have *Culicoides* hypersensitivity

b. Clinical allergy scores using a scoring range from 0 to 10 with scores ≥3 considered allergic (29).

c. Preclinical Phase occurred from April 12 – May 20 when there was allergen exposure but no clinical signs. Median and ranges are from a total of 9 scoring events per horse during the Preclinical Phase.

d. Allergic Phase occurred from May 24 – August 16 when individuals experienced consistent allergen exposure and showed clinical signs of allergy. Median and ranges are from a total of 11 scoring events per horse during the Allergic Phase.

e. Resolving Phase occurred from September 7 – November 1 when allergen exposure began to decrease and clinical signs started to improve. Median and ranges are from a total of 5 scoring events per horse during the Resolving Phase.

Icelandic horses in this study were either clinically healthy or had seasonal, recurrent *Cul* hypersensitivity. *Cul* hypersensitivity was confirmed, as previously described (19), by intradermal skin testing with *Cul* whole body extract (WBE; Stallergenes Greer Inc., Cambridge, MA, USA) in comparison to injections with saline and histamine as negative and positive controls, respectively. Allergic horses developed immediate reactions to *Cul* WBE, while healthy horses did not. Allergic horses (n=7) included 5 mares (12-18 years, median 17 years), 1 gelding (age 10) and 1 stallion (age 9). All 7 allergic horses and 6 of the healthy horses were also used in our previous study which characterized IgE+ plasmablasts in horses (17). The prior study took place in the summer and winter preceding the year of this study. Allergic horses had a history of seasonal *Cul* hypersensitivity for at least 3 years (median 8, range 3-8 years) before this study took place. Healthy horses (n=10) included 5 mares (ages 8-10 years, median 8 years) and 5 geldings (8-10 years, median 9 years).

Blood samples were obtained from the V. jugularis using the BD Vacutainer system (Becton Dickinson, Franklin Lakes, NJ). Samples were collected from all horses weekly from March 22-April 26, twice weekly from May 3-June 10, twice monthly from June 17- November 15, and once in February and December. Midges were first observed in the environment for a few days during the weeks of April 5 and April 19. April 26 was the last day in the spring when the temperature dropped to freezing temperature. Daily minimum and maximum temperatures were recorded from wunderground.com at a weather station <1 mile from the horse pasture.

At every blood collection day, clinical allergy scores were assigned to each horse as previously described (29). Scores were given based on pruritis (0-3), alopecia (0-4), and dermatitis (0-3) and total scores ≥3 represent horses with clinical allergy. Allergic horses developed clinical allergy and had clinical scores above 3 on average 15 timepoints (standard deviation 3.7) during the summer. Healthy horses never developed clinical signs or scores above 3. The first day when any allergic horse had clinical signs with a score above 3 was May 24.

All animal procedures were carried out in accordance with the recommendation in the Guide for the Care and Use of Laboratory Animals of the National Institutes of Health. The animal protocol was approved by the Institutional Animal Care and Use Committee at Cornell University (protocol #2011–0011). The study also followed the Guide for Care and Use of Animals in Agricultural Research and Teaching.

### 2.2 Isolation of peripheral blood leukocytes and acid wash approach to remove receptor-bound IgE

Heparinized blood samples settled at room temperature for at least 30 minutes to allow erythrocytes to separate from the cell-rich plasma. Cell rich plasma (1 ml), containing peripheral blood leukocytes (PBL), was collected and centrifuged at 500 xg for 10 minutes at 4°C. PBL were then treated with an acid wash as previously described (17). All other centrifugation steps were performed at 100 xg for 5 minutes at 4°C. Briefly, PBL were washed twice in ice-cold wash solution (130 mM NaCl, 5 mM KCl; pH 6). Cells were then resuspended in ice-cold acid wash solution (10 mM lactic acid (Alfa Aesar, Thermo Fisher Scientific, Lancashire, UK), 130 mM NaCl, 5 mM KCl; pH 2.8 – 3) and incubated for 5 min on ice. After incubation, 0.2 ml of 1 M Tris HCl, pH 8 (Thermo Fisher Scientific, Waltham, MA, USA) was added to neutralize the acid before pelleting the cells by centrifugation. Cells were washed once in phosphate buffered saline (PBS, Fisher Scientific, Waltham, MA, USA) and then fixed in 2% (v/v) paraformaldehyde solution (PFA, Sigma-Aldrich, St. Louis, MO, USA) for 20 minutes at room temperature.

In addition, peripheral blood mononuclear cells (PBMC) were isolated by layering cell-rich plasma over Ficoll (Ficoll Plaque Plus, GE Healthcare, Chicago, IL, USA) at a 2:1 (v:v) ratio. Density gradient centrifugation and PBMC isolation was performed as previously described (17). PBMC were also treated with the acid wash buffer as described for PBL samples.

### 2.3 Antibody staining and flow cytometry

For flow cytometric analysis of IgE+ plasmablasts, PBL or PBMC were stained as previously described (17). In brief, about 1.5x10^7^ PBL or 6x10^5^ PBMC were incubated for 15 minutes with an antibody master mix in PBS-BSA (0.5% (w/v) BSA, 0.02% (w/v) NaN3, all from Sigma-Aldrich, St. Louis, MO, USA). Three horse-specific monoclonal antibodies (mAbs) were used in the master mix: IgE mAb 176 (31) conjugated to Alexa fluorochrome 647, CD23 mAb 51-3 (32) conjugated to Alexa fluorochrome 488, and biotinylated IgG1 mAb CVS45 (33). Alexa conjugation and biotinylation of mAbs was performed according to manufacturer’s protocols (Thermo Fisher Scientific, Waltham, MA, USA). After incubation with the master mix, cells were washed once in PBS-BSA and then incubated with streptavidin-phycoerythrin (Jackson ImmunoResearch Laboratories) for 15 minutes to label biotinylated IgG1 mAb CVS45. IgG1 mAb and secondary labeling with streptavidin-phycoerythrin was included for another purpose. As previously described (17), IgE+ plasmablasts are IgG1- and therefore IgG1 data is not included here. Cells were washed in PBS-BSA one more time. Samples were recorded on a BD FACS Canto II flow cytometer and data analysis was performed with FlowJo version 10.4 (FlowJo, Ashland, OR, USA). A total of 150,000 events/sample were recorded for each sample. All flow cytometry images were gated first to exclude doublets, then IgE+ cells were analyzed quantitatively.

### 2.4 *In vitro* assay and measurement of total IgE secretion

Following the same PBL isolation as described above, 1 ml cell-rich plasma was collected and centrifuged at 500 xg for 10 minutes at 4°C. The cell-depleted plasma was collected and stored at -20°C until analyzed for IgE. PBL from 1 ml cell rich plasma were resuspended in cell culture medium (DMEM supplemented with 1% (v/v) non-essential amino acids, 2mM L-glutamine, 50µM 2-mercaptoethanol, 50µg/ml gentamicin, 100 U/ml penicillin, 100 µg/ml streptomycin (Thermo Fisher Scientific, Waltham, MA, USA) and 10% FCS (Atlanta biological, Flowery Branch, GA, USA)), and then centrifuged at 100 xg for 5 minutes at room temperature. PBL were resuspended to 1 ml in cell culture medium, and 100 µl of PBL from each individual was mixed with cell culture medium to a final volume of 200 µl and incubated in 96-well flat-bottomed plates (Corning Incorporated, Corning, NY, USA) at 37°C, 5% CO_2_. After 72-hours, cell-free supernatants were collected and stored at 4°C until IgE secretion analysis. All steps were performed in a sterile biosafety cabinet. PBMC were also isolated as described above and 5x10^5^ PBMC were plated per well in 200 µl cell culture medium. Cells were incubated as described above for PBL and supernatants were collected for IgE secretion analysis.

Secreted equine IgE was measured in undiluted supernatants using a fluorescent bead-based Luminex assay. The assay was set up as previously described (17, 34) with a few modifications. Twelve 1:2 serial dilutions of purified IgE were used as a standard with concentrations ranging from 4.9 ng/ml – 10 µg/ml total IgE. Total IgE was also measured in the cell-depleted plasma using eight 1:2 serial dilutions of purified IgE with concentrations ranging from 78.1 ng/ml – 10 µg/ml total IgE.

### 2.5 Statistical analysis

The data were not normally distributed as confirmed by D’Agostino and Pearson tests. Therefore, non-parametric tests were used for data analysis. To compare clinical scores, IgE+ plasmablast frequencies, CD23+ frequencies, and IgE concentrations between allergic and healthy horses at different timepoints, a Holm-Sidak multiple comparisons test was used. A nonparametric Spearman rank correlation was calculated for all horses to compare the frequency of IgE+ plasmablasts, total IgE+ cells after acid wash, secreted IgE concentrations, plasma IgE concentrations and clinical scores. All graphs plot means and standard deviations unless specified otherwise and p values <0.05 were considered significant. Analysis was performed with GraphPad Prism software version 8 (GraphPad Software Inc., La Jolla, CA, USA).

## 3 Results

### 3.1 Rapid identification of IgE+ plasmablasts in peripheral blood leukocytes

**Figure 1:**
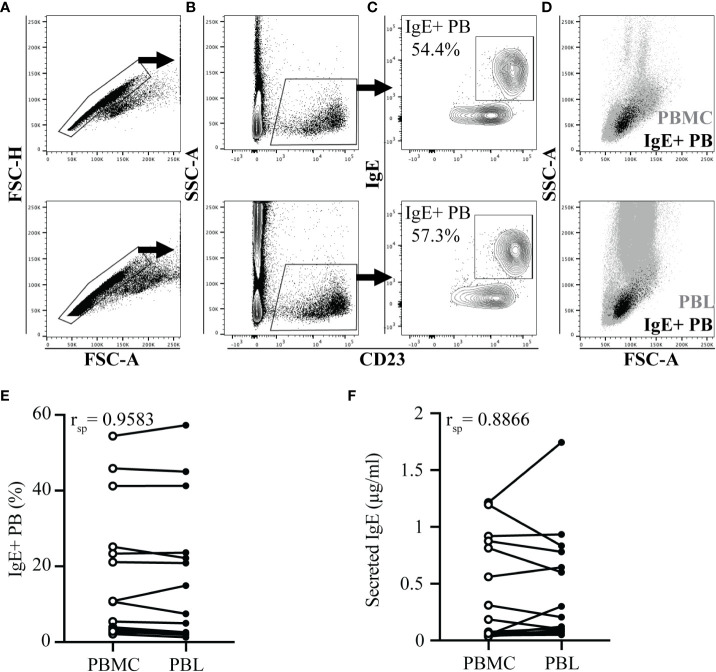
IgE^+^ plasmablast quantification in PBL. PBMC or PBL were isolated from heparinized whole blood of 17 horses on the same day. Blood collection occurred in the summer when allergic horses had clinical allergy and elevated peripheral IgE^+^ plasmablast frequencies. In A-D, PBMC samples are shown on the top and PBL samples are shown on the bottom. **(A)** Doublet exclusion. **(B)** CD23 expression gate on doublet excluded cells. **(C)** IgE and CD23 expression on CD23^+^ gated cells. Percentage reports IgE^+^ plasmablasts (IgE^+^ PB) out of total CD23^+^ cells. **(D)** Forward (FSC) and side scatter (SSC) characteristics of IgE^+^ plasmablasts (black) in total PBMC or PBL (gray). **(E)** Spearman rank correlation of the IgE^+^ plasmablast frequency in PBMC (open circles) and PBL (closed circles). **(F)** Spearman rank correlation of secreted IgE (µg/ml) in the supernatants of PBMC (open circles) and PBL (closed circles) after 72 hours *in vitro* culture. In **(E-F)**, samples from the same individual are connected. Flow cytometry images are representative from 1 out of 17 horses. Correlations were calculated with samples collected on July 19.

IgE+ plasmablasts were quantified in peripheral blood by a short lactic acid wash (pH 3), followed by incubation with fluorescent antibodies against IgE and CD23, and quantification by flow cytometry (17). To quantify the IgE+ plasmablast frequency more rapidly in many individuals, this approach was adapted from isolated PBMC (**Figures 1A-D** top images) to PBL (**Figures 1A-D** bottom images). PBL isolation only required erythrocyte removal or lysis. In both sample types, a similar gating strategy was used: doublets were excluded (**Figure 1A**), followed by gating on CD23+ cells (**Figure 1B**), and then on IgE+ cells (**Figure 1C**). IgE+ plasmablast identity was also confirmed by a larger forward (FSC) and side scatter (SSC), which is characteristic of these cells located between lymphocytes and monocytes in both PBMC and PBL (**Figure 1D**). The frequency of IgE+ plasmablasts out of total CD23+ cells was similar between PBMC and PBL samples in all samples (**Figure 1E**, n=17, r_sp_= 0.9583, p<0.0001). Spontaneous secretion of IgE after *in vitro* culture of PBMC or PBL for 72 hours also resulted in similar concentrations of IgE in the cell culture supernatants (**Figure 1F**, n=17, r_sp_= 0.8866, p<0.0001).

### 3.2 IgE+ plasmablast increase in peripheral blood precedes recurrent clinical allergy

To determine when IgE+ plasmablasts enter peripheral blood in comparison to the onset of clinical allergy, a group of allergic (n=7) and healthy horses (n=10) living under identical environmental conditions were monitored for one year. Heparinized blood was collected frequently from each horse (**Supplemental Figure 1**). Insects were first observed in the environment of the horses the week of April 5, and were consistently present after April 26, which was the last night with temperatures below freezing.

**Figure 2:**
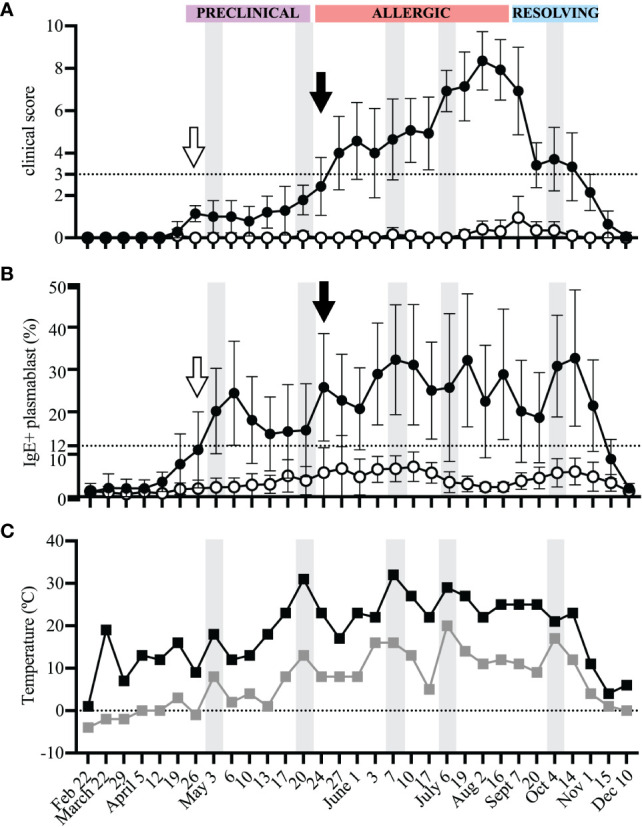
IgE^+^ plasmablasts appear in peripheral blood before the onset of clinical allergy. PBL were frequently collected from heparinized blood samples for the duration of one year to compare the onset of IgE+ plasmablasts in peripheral blood and the development of clinical allergy. Samples were collected from allergic (n=7, black circles) and healthy (n=10, open circles) horses **(A)** Clinical scores (0-10) were assigned to each horse at each timepoint using the Cornell allergy scoring system described by Miller et al. (29). The dotted horizontal line denotes the threshold ≥3 where allergic horses have clinical allergy. The three phases of the allergen exposure season (Preclinical, Allergic and Resolving) are labeled in purple, red and blue boxes, respectively. **(B)** The frequency of IgE^+^ plasmablasts was measured at each timepoint. The dotted horizontal line represents the threshold were allergic horses have ≥12% IgE^+^ plasmablasts. **(C)** Daily minimum (gray squares) and maximum (black squares) temperatures (°C) were recorded at each blood sampling timepoint. **(A-C)** Gray vertical bars show high temperature peaks (May 3, May 20, June 7, July 6, October 4). **(A, B)** Open arrows (April 26) show the first day of IgE^+^ plasmablasts above the threshold of 12% in allergic horses. Black arrows (May 24) show first day when clinical allergy scores above the threshold of 3 were given to allergic horses. Graphs show mean and standard deviation.

At each sampling day, horses were assigned clinical scores to quantify signs of allergy (**Figure 2A**). The season of allergen exposure was split into three phases: (1) “Preclinical Phase” when allergen exposure began but there were no clinical signs (timepoints from April 12-May 20), (2) “Allergic Phase” when individuals experienced continuous allergen exposure and clinical disease (timepoints from May 24 – Aug 16), and (3) “Resolving Phase” when allergen exposure began to decrease and individuals started to resolve their clinical signs (timepoints from Sept 7 – Nov 1) (**Figure 2A**).

**Figure 3:**
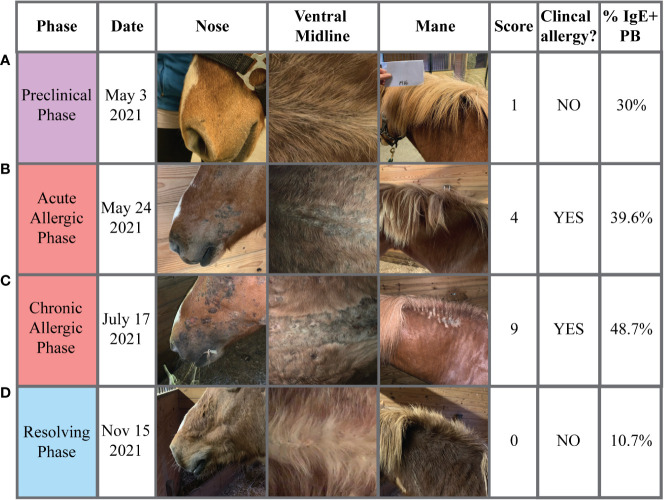
Progression of clinical signs of allergy. Photographs of each horse were taken to document changes in clinical signs of allergy (*Cul* hypersensitivity) at each phase of allergen exposure (Preclinical, Allergic, Resolving). Pictures from one allergic horse with common skin lesion and hair loss sites at the face/nose, ventral midline and mane/neck are shown. The clinical allergy score (0-10), whether the signs were considered clinical allergy (yes/no), and IgE+ plasmablast frequencies (%) were also reported for each timepoint. **(A)** The Preclinical Phase (May 3) when insects and midges were present in the environment and IgE^+^ plasmablasts were above the threshold of 12% in allergic horses. **(B)** The start of the Allergic Phase (May 24) when allergic horses first started to develop acute clinical disease. **(C)** The middle of the Allergic Phase (June 17) when allergic horses were experiencing consistent allergen exposure, sustained IgE^+^ plasmablast frequencies in peripheral blood, and severe chronic clinical disease. **(D)** The end of the Resolving Phase (November 15) when midges had died and were no longer present in the environment and all allergic horses had clinically recovered. Pictures are representative from one of seven allergic horses.

Photographs were taken of each horse to further document the changes in clinical signs of allergy throughout the different phases of allergen exposure (**Figure 3**). During the “Preclinical Phase” in early May, all horses still looked clinically healthy (**Figure 3A**). Some allergic horses (2 out of 7) showed first clinical signs of allergy on May 24 (**Figure 2A**, black arrow, **Figure 3B**), marking the start of the “Allergic Phase”, and the majority (5 out of 7) showed clinical signs on May 27. Clinical signs worsened in all allergic horses throughout the "Allergic Phase" (**Figure 3C**). The allergic horses entered the "Resolving Phase" after all midges died in the first frost on Nov 1 (**Figure 3D**).

At each timepoint, IgE+ plasmablasts were quantified by flow cytometry of acid wash treated PBL (**Figure 2B**). A threshold of 12% IgE+ plasmablasts (out of total CD23+ cells) differentiates allergic from healthy horses as previously described (17). In allergic horses, IgE+ plasmablast frequencies began to rise above the threshold during the “Preclinical Phase” on April 26 (2 out of 7 horses, **Figure 2B**, open arrow) and the majority (6 out of 7) were above the 12% threshold on May 3. This increase in IgE+ plasmablasts in the peripheral blood preceded development of clinical allergy (May 24, **Figures 2A, B**, black arrows) by 3 weeks. One allergic horse had a delayed onset of clinical allergy with the first score above 3 on July 6. In this horse, IgE+ plasmablast frequency exceeded 12% on June 1. In summary, these data strongly demonstrate that IgE+ plasmablasts enter the circulation at least 3 weeks before the onset of recurrent clinical allergy.

The frequency of IgE+ plasmablasts was maintained in allergic horses above the threshold of 12% for the duration of the summer. IgE+ plasmablasts increased to over 40% in 3 out of 7 horses during the “Allergic Phase”, with one horse reaching as high as 57% IgE+ plasmablasts. The frequency of IgE+ plasmablasts also fluctuated with changes in weather. Daily minimum and maximum temperatures were recorded on every sampling day for the duration of the study (**Figure 2C**). Increases in environmental temperatures generally support the activity of *Cul* midges. Five warm weather peak days (gray vertical bars on May 3, May 20, June 7, July 6, October 4, **Figure 2C**) were followed by IgE+ plasmablast peaks on the next sampling timepoints (May 6, May 24, June 10, July 19, October 14, **Figure 2B**). Circulating IgE+ plasmablasts, therefore, are responsive to environmental changes, which can be used as a surrogate measurement for midge burden and allergen exposure.

### 3.3 Peripheral blood IgE+ plasmablasts are rare cells

**Figure 4:**
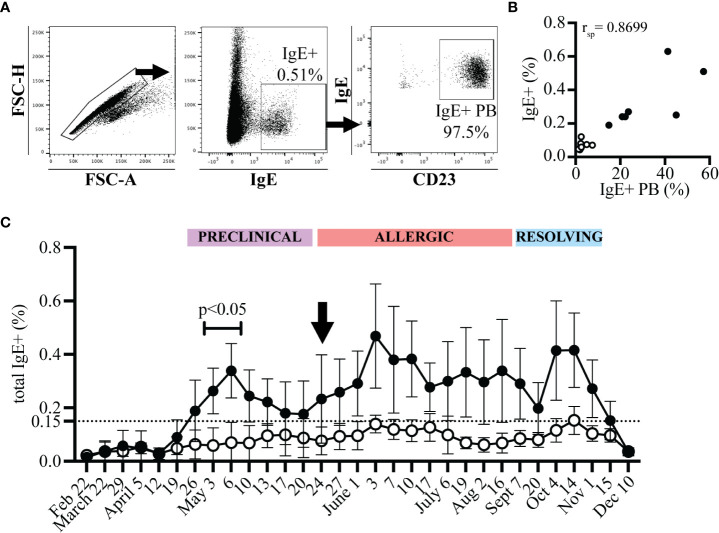
IgE+ plasmablasts are detectable in total PBL samples by acid wash followed by anti-IgE staining only. PBL were collected at each timepoint from allergic (n=7, black circles) and healthy (n=10, open circles) horses. PBL were acid washed and stained with an antibody against IgE. **(A)** Gating strategy to quantify IgE+ plasmablasts by only IgE expression. Doublets were excluded (left), IgE+ cells were selected (middle), and expression of CD23 by IgE+ cells was confirmed. **(B)** Spearman rank correlation of the frequency of IgE+ plasmablasts (IgE+ out of CD23+) and of total IgE+ cells (out of PBL) on July 19 from allergic (n=7, black circles) and healthy (n=10, open circles) horses. **(C)** The frequency of total IgE+ cells at each timepoint. The dotted line represents the threshold were allergic horses have ≥0.15% total IgE+ cells a few weeks prior to and during clinical allergy. Flow cytometry images are representative from 1 out of 17 horses. Graphs show mean and standard deviation.

Alternatively, IgE+ plasmablasts can be measured with only one antibody, against IgE, after acid wash treatment (17). Acid wash treatment removed all receptor-bound IgE and therefore the remaining IgE+ cells represented IgE+ plasmablasts (**Figure 4A**). The frequency of IgE+ plasmablasts measured as total IgE+ cells or out of CD23+ cells strongly correlated (**Figure 4B**, n=17, r_sp_=0.8699, p<0.0001, on July 19). The frequency of IgE+ cells out of total PBL after acid treatment also described the increase in IgE+ plasmablasts that occurred during the “Preclinical Phase” on April 26 (**Figure 4C**). On April 12, all horses had total IgE+ frequencies <0.04%. However, on April 26 this frequency increased more than 4-fold in allergic horses to 0.19% (mean, SD 0.115) while remaining below 0.06% in healthy horses. IgE+ cells reached as high as 0.65% of total PBL in one allergic horse on June 3. A threshold of 0.15% IgE+ plasmablasts (out of total PBL) differentiated allergic from healthy horses. This further highlighted the rapid influx of IgE+ plasmablasts into the circulation before the onset of clinical signs and provided a simplified method of IgE+ plasmablast detection in peripheral blood.

### 3.4 IgE secretion by IgE+ plasmablasts correlates with disease severity

**Figure 5:**
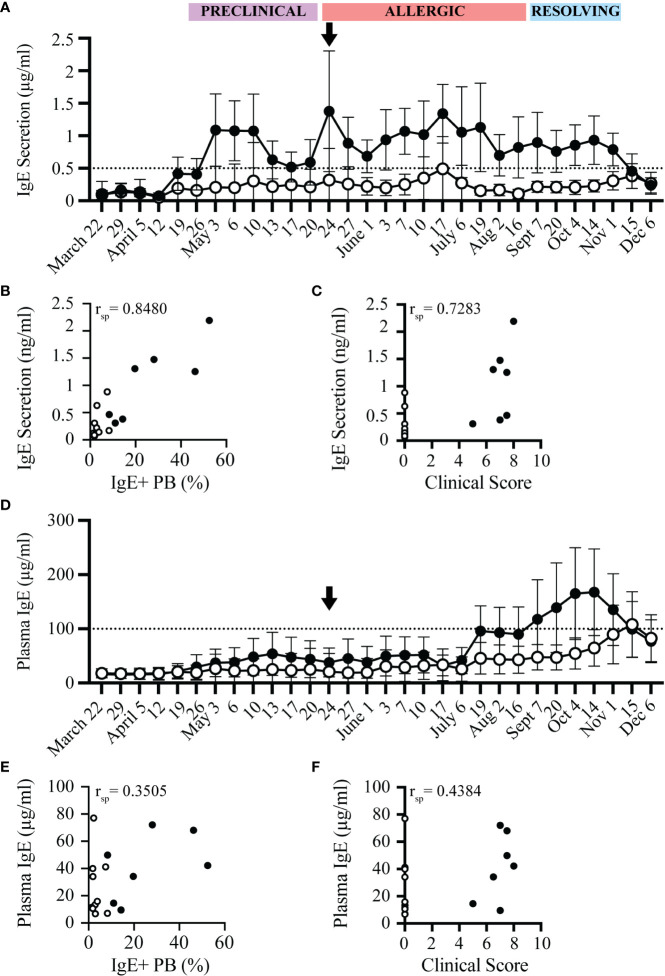
IgE secretion from PBL, but not plasma IgE, increases before onset of clinical allergy. PBL and cell-depleted plasma samples were collected from allergic (n=7, black circles) and healthy (n=10, open circles) horses every 3-14 days for one year. The concentration of secreted IgE by PBL was evaluated in cell culture supernatants after 72 hours of *in vitro* culture. IgE concentrations were measured by a bead-based IgE assay. **(A)** Secreted IgE was compared at each timepoint. The dotted line denotes the threshold where allergic horses had ≥ 0.5 µg/ml secreted IgE. Black arrow shows the first day allergic horses had clinical allergy scores above the clinical threshold (May 24). **(B)** Spearman rank correlation of the frequency of IgE+ plasmablasts (out of CD23+) and the concentration of secreted IgE from PBL (µg/ml). **(C)** Spearman rank correlation of clinical score and the concentration of secreted IgE from PBL (µg/ml). **(D)** Total IgE (µg/ml) in plasma was compared at each timepoint. The dotted line denotes the threshold where allergic horses had ≥ 100 µg/ml plasma IgE. **(E)** Spearman rank correlation of the frequency of IgE+ plasmablasts (out of CD23+) and the concentration of plasma IgE (µg/ml). **(F)** Spearman rank correlation of clinical score and the concentration of plasma IgE (µg/ml). All correlations were calculated with samples collected on July 6, during the Chronic Phase.

IgE+ plasmablasts spontaneously secrete IgE (17). IgE secretion by these cells was measured after 72 hours of *in vitro* culture of PBL. The concentration of secreted IgE from allergic and healthy horses followed a similar trend as the frequency of IgE+ plasmablasts (**Figure 5A**). IgE secretion increased in allergic horses at the beginning of the “Preclinical Phase” on April 26, and was maintained above a threshold of 0.5 µg/ml for the duration of the summer. In general, IgE secretion by PBL from healthy horses stayed below 0.5 µg/ml. However, occasionally a healthy horse had elevated IgE secretion. In these instances, the frequency of IgE+ plasmablasts were also elevated by flow cytometry. Overall, spontaneous IgE secretion from PBL correlated positively with IgE+ plasmablast frequencies (r_sp_=0.8480, p<0.0001, **Figure 5B**) and clinical allergy scores (r_sp_=0.7283, p<0.005, **Figure 5C**). IgE secretion can thus serve as another method to quantify IgE+ plasmablasts and predict onset of allergy.

In comparison to the spontaneous IgE secretion from PBL, total IgE concentrations in plasma from the same blood samples were also measured in all horses. Total IgE in plasma was only elevated in some allergic individuals later in the summer, at the end of the “Allergic Phase” and peaked during the “Resolving Phase”. On July 17, 4 out of 7 allergic horses had plasma IgE concentrations above the plasma IgE threshold of 100 µg/ml. Plasma IgE in 2 of the allergic horses did not cross the threshold until the “Resolving Phase” in October, 5 months after clinical allergy onset. One horse never reached the plasma IgE threshold (**Figure 5D**). Plasma IgE concentrations, therefore, had a weak correlation with IgE+ plasmablast frequencies (r_sp_=0.3505, p=0.1681, **Figure 5E**) and clinical allergy scores (r_sp_=0.4384, p=0.0797, **Figure 5F**). In summary, the increase of IgE concentrations in plasma is delayed by at least 8 weeks (2 months) after the onset of clinical allergy, does not distinguish allergic from healthy horses before or during the onset of allergy, and cannot be used to predict disease. All raw data is included in **Supplemental Figure 2**.

## 4 Discussion

IgE is the essential link between effector cells and allergen to cause IgE-mediated allergic diseases. Therefore, the cells that produce allergen-specific IgE are critical for the maintenance of allergy. IgE+ plasmablasts are a cell type in the lineage of IgE+ B cells (15). Recently, we described that IgE+ plasmablasts are circulating in the peripheral blood and are increased in allergic individuals during clinical allergy (17). Here, we described the longitudinal relationship of IgE+ plasmablasts with allergen exposure by frequently surveying their presence in peripheral blood before, during and after allergen exposure. We found that IgE+ plasmablasts are sensitive indicators of allergen exposure by entering in the peripheral blood almost immediately following the presence of allergen in the environment. We also discovered that the appearance of IgE+ plasmablasts in the circulation preceded clinical allergy by at least three weeks in a seasonal allergy model of IgE-mediated *Cul* hypersensitivity.

IgE+ plasmablasts, therefore, can serve as an immediate snapshot of allergen-specific B cell re-activation (recall response) after allergen exposure. In our study, allergen exposure was dependent on the outdoor temperature, where warmer humid days have higher midge burden, and therefore higher allergen exposure through midge bites. We found that the frequency of IgE+ plasmablasts increased after each peak in warm weather, showing that IgE+ plasmablasts rapidly develop and enter circulation following allergen exposure and thereby continue to promote clinical signs of allergy.

Currently, detection of allergen-specific IgE in serum, *via* ELISA, or testing sensitization of mast cells with allergen-specific IgE, *via* skin prick tests, are the primary means to diagnose and monitor allergies (2–4). Both tests are dependent on the secretion of allergen-specific IgE by plasma cells and are typically used after clinical allergy developed. In contrast, IgE+ plasmablasts are a source of IgE and can distinguish allergic individuals early and before clinical disease onset. IgE+ plasmablasts provide a sensitive blood marker to directly measure the response to recent allergen contact in an individual. Our data presented here therefore support that the measurement of IgE+ plasmablasts can be used as a novel biomarker of increased IgE production and allergen sensitization. Based on the close correlation between allergen exposure and increasing IgE+ plasmablast frequencies in allergic individuals, it can also be hypothesized that IgE+ plasmablasts decrease following successful allergen immunotherapy injection and would provide a biomarker to support treatment efficacy by desensitizing the patient. This treatment-related biomarker function of IgE+ plasmablasts, however, still requires confirmation by future studies.

In addition, IgE+ plasmablasts can also be measured by their IgE secretion, and we showed that this IgE secretion also distinguishes allergic from healthy individuals. This contrasted with measurement of total plasma IgE concentrations. We showed that, in horses, plasma IgE levels do not distinguish allergic horses until later in the summer during chronic allergy, and even then, not all allergic individuals exhibit elevated plasma IgE. There are multiple explanations why the influx of IgE+ plasmablasts, which secrete IgE, do not always result in an increase in plasma IgE. First, secreted IgE by plasmablasts may rapidly bind to IgE receptors and therefore not contribute to the plasma IgE concentration. Second, IgE+ plasmablasts may be short lived (15) or only circulate transiently before further migration and differentiation, which would also limit the accumulation of plasma IgE. Third, the serum or plasma IgE concentration at any given time is less dynamic due to the constant production of IgE by resident plasma cells in lymphatic tissues. This has been shown in humans, where IgE-secreting cells in blood were responsible for producing less than 1% of serum IgE (35). As a result, measuring IgE+ plasmablasts, either directly by flow cytometry or indirectly by IgE secretion, provides earlier and more accurate information of the recent allergen activation occurring in an individual.

While there are candidate biomarkers to diagnose pre-existing allergies (3), predictors of allergy development before the onset of clinical signs do not currently exist. A recent meta-analysis explored potential predictive biomarkers of allergy development in infants, but these have not yet been further explored or validated (36). However, plasmablasts in other diseases, such as ulcerative colitis, can predict future disease severity (37), suggesting that plasmablasts in allergy may provide a similar biomarker capability.

This study was performed in horses already allergic for 3-8 years with a naturally occurring, seasonal IgE-mediated allergy called *Cul* hypersensitivity (26, 27). Our data support that IgE+ plasmablasts, as part of the recall response to allergen, predict development of recurrent clinical allergy. Clinical allergy developed about a month after allergen exposure begin. During the “Preclinical Phase”, the 3-4 week period when allergen exposure began, we found that IgE+ plasmablasts increased dramatically in allergic horses while they still looked clinically healthy. In humans with seasonal pollen allergies, the time between allergen exposure and symptom development is shorter (38, 39). However, low pollen levels also occur in the weeks prior to the “pollen allergy season” (40). It is therefore likely that in humans with seasonal allergies, IgE+ plasmablasts enter circulation at the first annual exposure to low pollen levels before the annual onset of clinical disease.

While IgE+ plasmablasts have been identified in humans using complex staining and identification procedures (41–44), they have not been measured with a lactic acid wash, as described here, or studied in relationship with recent allergen exposure. The equine IgE-mediated allergic immune response is mechanistically similar to that in allergic humans (18, 45), and we have previously shown that IgE+ plasmablasts are conserved between humans and horses (17). Prior studies also suggest that human IgE+ plasmablasts exhibit similar behavior following allergen exposure. Humans with atopic dermatitis have elevated IgE+ plasmablasts in peripheral blood compared to healthy controls (41, 44). Additionally, in humans with seasonal allergy, these IgE+ cells are present in peripheral blood only during allergen exposure (46). This further supports a conserved function of IgE+ plasmablasts during allergy development and maintenance across mammalian species with naturally occurring IgE-mediated allergies. Therefore, it is likely that IgE+ plasmablasts also have a predictive biomarker capacity in allergic humans or other veterinary species, such as dogs and cats.

IgE-mediated hypersensitivities begin in a silent sensitization phase, when allergen-specific B cells are activated but there are no clinical signs. Following subsequent allergen exposure, IgE-switched memory cells, and IgE+ plasmablasts, are then activated. While this study shows the reactivation of B cells in individuals with pre-existing allergy, we have shown here for the first time that the appearance of new IgE+ plasmablasts in peripheral blood happens in the critical window between a new allergen exposure and before the onset of clinical signs of allergy.

In addition to serving as a predictive biomarker of allergy, IgE+ plasmablasts also provide insight into the mechanism of allergen-specific IgE production and maintenance in an individual. There are two mechanisms of IgE class switching in mice, directly from IgM to IgE or sequentially through an intermediate class switching to IgG1 (47, 48). Sequential class switching is essential for the development of high-affinity IgE (49) and differentiation into IgE+ plasma cells (50). The same is likely true in humans and horses. A recent study measured human IgE+ plasmablasts by single cell sequencing and found that the IgE specificity was identical to the clonotypes of IgG+ B cells in the same individual (51), supporting that IgE+ plasmablasts have undergone sequential class switching through IgG. These IgG and IgE clones, with identical antigen specificity, also expressed CD23 transcripts (51), providing further evidence that they were similar to the IgE+ plasmablasts described in this study. In addition, this study found that IgE+ plasmablasts rapidly disappeared when allergen exposure ceased (51), suggesting that IgE+ plasmablasts are either short-lived cells (52, 53) or quickly migrate into the bone marrow to differentiate into IgE secreting plasma cells (54, 55).

Sequential class switching to IgE can occur both in germinal centers and also in local tissue-associated lymphatic tissues (41, 56). It is possible, then, that IgE+ plasmablasts are developed in close proximity of allergen exposure in the tissue-associated lymphatic tissues, such as the skin, instead of the local lymph node. A recent study of IgE class switching in the nasal mucosa of allergic rhinitis patients found that sequentially switched IgE+ plasmablasts were present in both the nasal mucosa and peripheral blood following nasal allergen exposure (57). In addition, the authors were able to detect class switch circles in the nasal mucosa, which are generated only during class switch recombination, suggesting local class switching and subsequent migration of IgE+ plasmablasts into the periphery (57).

What happens to IgE+ plasmablasts following allergen exposure also remains to be explored. We have shown that IgE+ plasmablasts exist transiently in peripheral blood and are maintained by recurrent allergen exposure. Plasmablasts have also been studied in other diseases, including viral infection (58) and Lupus Erythematosus (59), providing clues to how plasmablasts may act during allergy. Most likely, IgE+ plasmablasts are short lived cells, as has been shown in the context of both malaria-induced (52) and allergy-induced (15) plasmablasts. Plasmablasts in allergic humans, analyzed by single cell RNA sequencing, downregulated SYK expression (15), which is essential for B cell survival and differentiation. It will be important for future work to further describe the cell fate that is adopted by IgE+ plasmablasts.

In conclusion, we have identified IgE+ plasmablasts as a novel peripheral blood biomarker of allergy that precedes recurrent clinical allergy, is rapidly responsive to changes in allergen exposure, and is easily identifiable after an acid wash approach. We propose IgE+ plasmablasts as a predictive biomarker during allergen sensitization before first onset of disease, and as a treatment success marker during allergen immunotherapy treatment.

## Data availability statement

The original contributions presented in the study are included in the article/**Supplementary Material**. Further inquiries can be directed to the corresponding author.

## Ethics statement

The animal study was reviewed and approved by Institutional Animal Care and Use Committee at Cornell University.

## Author contributions

Conceptualization and methodology were designed by ES and SB. ES, SB, and JT performed experiments. Formal analysis, writing, and editing were all done by ES, SB, and BW. Funds were acquired by BW. All authors contributed to the article and approved the submitted version.

## References

[1] Galli SJ Tsai M. IgE and mast cells in allergic disease. Nat Med (2012) 18:693–704. doi: 10.1038/nm.2755 22561833 PMC3597223

[2] Ciprandi G Tosca MA Silvestri M. The practical role of serum allergen-specific IgE as potential biomarker for predicting responder to allergen immunotherapy. Expert Rev Clin Immunol (2014) 10:321–4. doi: 10.1586/1744666X.2014.872032 24450987

[3] Muraro A Arasi S. Biomarkers in food allergy. Curr Allergy Asthma Rep (2018) 18:64. doi: 10.1007/s11882-018-0816-4 30284049

[4] Letrán A Espinazo M Moreno F. Measurement of IgE to pollen allergen components is helpful in selecting patients for immunotherapy. Ann Allergy Asthma Immunol (2013) 111:295–7. doi: 10.1016/j.anai.2013.07.005 24054367

[5] Hazebrouck S Guillon B Paty E Dreskin SC Adel-Patient K Bernard H. Variable IgE cross-reactivity between peanut 2S-albumins: The case for measuring IgE to both ara h 2 and ara h 6. Clin Exp Allergy (2019) 49:1107–15. doi: 10.1111/cea.13432 PMC6684383 31108010

[6] Ehlers AM Blankestijn MA Knulst AC Klinge M Otten HG. Can alternative epitope mapping approaches increase the impact of b-cell epitopes in food allergy diagnostics? Clin Exp Allergy (2019) 49:17–26. doi: 10.1111/cea.13291 30294841 PMC7380004

[7] Bousquet J Rabe K Humbert M Chung KF Berger W Fox H. Predicting and evaluating response to omalizumab in patients with severe allergic asthma. Respir Med (2007) 101:1483–92. doi: 10.1016/j.rmed.2007.01.011 17339107

[8] Finkelman FD Katona IM Urban JF Holmes J Ohara J Tung AS. IL-4 is required to generate and sustain *in vivo* IgE responses. J Immunol (1988) 141:2335–41. doi: 10.4049/jimmunol.141.7.2335 2459206

[9] Lebman DA Coffman RL. Interleukin 4 causes isotype switching to IgE in T cell-stimulated clonal b cell cultures. J Exp Med (1988) 168:853–62. doi: 10.1084/jem.168.3.853 PMC2189023 3049907

[10] Punnonen J Aversa G Cocks BG McKenzie AN Menon S Zurawski G. Interleukin 13 induces interleukin 4-independent IgG4 and IgE synthesis and CD23 expression by human b cells. Proc Natl Acad Sci USA (1993) 90:3730–4. doi: 10.1073/pnas.90.8.3730 PMC46375 8097323

[11] Wagner B Siebenkotten G Radbruch A Leibold. Nucleotide sequence W. And restriction fragment length polymorphisms of the equine cvarepsilon gene. Vet Immunol Immunopathol (2001) 82:193–202. doi: 10.1016/s0165-2427(01)00355-5 11587734

[12] Looney TJ Lee J-Y Roskin KM Hoh RA King J Glanville J. Human b-cell isotype switching origins of IgE. J Allergy Clin Immunol (2016) 137:579–586.e7. doi: 10.1016/j.jaci.2015.07.014 26309181 PMC4747810

[13] Erazo A Kutchukhidze N Leung M Christ APG Urban JF Curotto de Lafaille MA. Unique maturation program of the IgE response *in vivo* . Immunity (2007) 26:191–203. doi: 10.1016/j.immuni.2006.12.006 17292640 PMC1892589

[14] Yang Z Robinson MJ Chen X Smith GA Taunton J Liu W. Regulation of b cell fate by chronic activity of the IgE b cell receptor. eLife (2016) 5:e21238. doi: 10.7554/eLife.21238 27935477 PMC5207771

[15] Croote D Darmanis S Nadeau KC Quake SR. High-affinity allergen-specific human antibodies cloned from single IgE b cell transcriptomes. Science (2018) 362:1306–9. doi: 10.1126/science.aau2599 30545888

[16] Nutt SL Hodgkin PD Tarlinton DM Corcoran LM. The generation of antibody-secreting plasma cells. Nat Rev Immunol (2015) 15:160–71. doi: 10.1038/nri3795 25698678

[17] Simonin EM Babasyan S Wagner B. Peripheral CD23hi/IgE+ plasmablasts secrete IgE and correlate with allergic disease severity. J Immunol (2022) 209:1–10. doi: 10.4049/jimmunol.2101081 35896336

[18] Larson EM Wagner B. Viral infection and allergy – what equine immune responses can tell us about disease severity and protection. Mol Immunol (2021) 135:329–41. doi: 10.1016/j.molimm.2021.04.013 33975251

[19] Wagner B Miller WH Morgan EE Hillegas JM Erb HN Leibold W. IgE and IgG antibodies in skin allergy of the horse. Vet Res (2006) 37:813–25. doi: 10.1051/vetres:2006039 16973120

[20] Anderson GS Belton P Kleider N. Hypersensitivity of horses in British Columbia to extracts of native and exotic species of culicoides (Diptera: Ceratopogonidae). J Med Entomol (1993) 30:657–63. doi: 10.1093/jmedent/30.4.657 8360890

[21] Braverman Y. Preferred landing sites of culicoides species (Diptera: Ceratopogonidae) on a horse in Israel and its relevance to summer seasonal recurrent dermatitis (sweet itch). Equine Vet J (1988) 20:426–9. doi: 10.1111/j.2042-3306.1988.tb01566.x 3215168

[22] Greiner EC Fadok VA Rabin EB. Equine culicoides hypersensitivity in Florida: biting midges aspirated from horses. Med Vet Entomol (1990) 4:375–81. doi: 10.1111/j.1365-2915.1990.tb00454.x 2133005

[23] Larsen H Bakke S Mehl R. Intradermal challenge of icelandic horses in Norway and Iceland with extracts of culicoides spp. Acta Vet Scand (1988) 29:311–4. doi: 10.1186/BF03548623 PMC8161629 3256230

[24] Steinman A Peer G Klement E. Epidemiological study of culicoides hypersensitivity in horses in Israel. Vet Rec (2003) 152:748–51. doi: 10.1136/vr.152.24.748 12833935

[25] Wagner B Childs BA Erb HN. A histamine release assay to identify sensitization to culicoides allergens in horses with skin hypersensitivity. Vet Immunol Immunopathol (2008) 126:302–8. doi: 10.1016/j.vetimm.2008.09.001 18926574

[26] van der Meide NMA Roders N Sloet van Oldruitenborgh-Oosterbaan MM Schaap PJ van Oers MM Leibold W. Cloning and expression of candidate allergens from culicoides obsoletus for diagnosis of insect bite hypersensitivity in horses. Vet Immunol Immunopathol (2013) 153:227–39. doi: 10.1016/j.vetimm.2013.03.005 23561552

[27] Schaffartzik A Marti E Torsteinsdottir S Mellor PS Crameri R Rhyner C. Selective cloning, characterization, and production of the culicoides nubeculosus salivary gland allergen repertoire associated with equine insect bite hypersensitivity. Vet Immunol Immunopathol (2011) 139:200–9. doi: 10.1016/j.vetimm.2010.10.015 21071100

[28] Wagner B Miller WH Erb HN Paul Lunn D Antczak DF. Sensitization of skin mast cells with IgE antibodies to culicoides allergens occurs frequently in clinically healthy horses. Vet Immunol Immunopathol (2009) 132:53–61. doi: 10.1016/j.vetimm.2009.09.015 19836083

[29] Miller JE Mann S Fettelschoss-Gabriel A Wagner B. Comparison of three clinical scoring systems for culicoides hypersensitivity in a herd of icelandic horses. Vet Derm (2019) 30:536–e163. doi: 10.1111/vde.12784 31441172

[30] Raza F Ivanek R Freer H Reiche D Rose H Torsteinsdóttir S. Cul o 2 specific IgG3/5 antibodies predicted culicoides hypersensitivity in a group imported icelandic horses. BMC Vet Res (2020) 16:283. doi: 10.1186/s12917-020-02499-w 32778104 PMC7418374

[31] Wagner B Radbruch A Rohwer J Leibold W. Monoclonal anti-equine IgE antibodies with specificity for different epitopes on the immunoglobulin heavy chain of native IgE. Vet Immunol Immunopathol (2003) 92:45–60. doi: 10.1016/S0165-2427(03)00007-2 12628763

[32] Wagner B Hillegas JM Babasyan S. Monoclonal antibodies to equine CD23 identify the low-affinity receptor for IgE on subpopulations of IgM+ and IgG1+ b-cells in horses. Vet Immunol Immunopathol (2012) 146:125–34. doi: 10.1016/j.vetimm.2012.02.007 22405681

[33] Sheoran AS Lunn DP Holmes MA. Monoclonal antibodies to subclass-specific antigenic determinants on equine immunoglobulin gamma chains and their characterization. Vet Immunol Immunopathol (1998) 62:153–65. doi: 10.1016/S0165-2427(97)00162-1 9638859

[34] Larson EM Babasyan S Wagner B. Phenotype and function of IgE-binding monocytes in equine culicoides hypersensitivity. PloS One (2020) 15:e0233537. doi: 10.1371/journal.pone.0233537 32442209 PMC7244122

[35] Eckl-Dorna J Pree I Reisinger J Marth K Chen K-W Vrtala S. The majority of allergen-specific IgE in the blood of allergic patients does not originate from blood-derived b cells or plasma cells. Clin Exp Allergy (2012) 42:1347–55. doi: 10.1111/j.1365-2222.2012.04030.x 22925321

[36] Davis EC Jackson CM Ting T Harizaj A Järvinen KM. Predictors and biomarkers of food allergy and sensitization in early childhood. Ann Allergy Asthma Immunol (2022) 129:292–300. doi: 10.1016/j.anai.2022.04.025 35490857 PMC11910167

[37] Uzzan M Martin JC Mesin L Livanos AE Castro-Dopico T Huang R. Ulcerative colitis is characterized by a plasmablast-skewed humoral response associated with disease activity. Nat Med (2022) 28:766–79. doi: 10.1038/s41591-022-01680-y PMC9107072 35190725

[38] de Weger LA Beerthuizen T Gast-Strookman JM van der Plas DT Terreehorst I Hiemstra PS. Difference in symptom severity between early and late grass pollen season in patients with seasonal allergic rhinitis. Clin Transl Allergy (2011) 1:18. doi: 10.1186/2045-7022-1-18 22410160 PMC3339365

[39] de Weger LA Hiemstra PS Op den Buysch E van Vliet AJH. Spatiotemporal monitoring of allergic rhinitis symptoms in the Netherlands using citizen science. Allergy (2014) 69:1085–91. doi: 10.1111/all.12433 24888457

[40] Lo F Bitz CM Battisti DS Hess JJ. Pollen calendars and maps of allergenic pollen in north America. Aerobiol (Bologna) (2019) 35:613–33. doi: 10.1007/s10453-019-09601-2 PMC6934246 31929678

[41] Berkowska MA Heeringa JJ Hajdarbegovic E van der Burg M Thio HB van Hagen PM. Human IgE+ b cells are derived from T cell–dependent and T cell–independent pathways. J Allergy Clin Immunol (2014) 134:688–697.e6. doi: 10.1016/j.jaci.2014.03.036 24835500

[42] Jiménez-Saiz R Ellenbogen Y Bruton K Spill P Sommer DD Lima H. Human BCR analysis of single-sorted, putative IgE+ memory b cells in food allergy. J Allergy Clin Immunol (2019) 144:336–339.e6. doi: 10.1016/j.jaci.2019.04.001 30959060 PMC7010227

[43] Ramadani F Upton N Hobson P Chan Y-C Mzinza D Bowen H. Intrinsic properties of germinal center-derived b cells promote their enhanced class switching to IgE. Allergy (2015) 70:1269–77. doi: 10.1111/all.12679 PMC4744720 26109279

[44] Heeringa JJ Rijvers L Arends NJ Driessen GJ Pasmans SG van Dongen JJM. IgE-expressing memory b cells and plasmablasts are increased in blood of children with asthma, food allergy, and atopic dermatitis. Allergy (2018) 73:1331–6. doi: 10.1111/all.13421 29380876

[45] Horohov DW. The equine immune responses to infectious and allergic disease: A model for humans? Molec Immunol (2015) 66:89–96. doi: 10.1016/j.molimm.2014.09.020 25457878

[46] Eckl-Dorna J Villazala-Merino S Campion NJ Byazrova M Filatov A Kudlay D. Tracing IgE-producing cells in allergic patients. Cells (2019) 8:994. doi: 10.3390/cells8090994 31466324 PMC6769703

[47] Matsuoka M Yoshida K Maeda T Usuda S Sakano H. Switch circular DNA formed in cytokine-treated mouse splenocytes: Evidence for intramolecular DNA deletion in immunoglobulin class switching. Cell (1990) 62:135–42. doi: 10.1016/0092-8674(90)90247-C 2114219

[48] Saunders SP Ma EGM Aranda CJ Curotto de Lafaille MA. Non-classical b cell memory of allergic IgE responses(2019) (Accessed June 22, 2022). 10.3389/fimmu.2019.00715 PMC6498404 31105687

[49] Xiong H Dolpady J Wabl M Curotto de Lafaille MA Lafaille JJ. Sequential class switching is required for the generation of high affinity IgE antibodies. J Exp Med (2012) 209:353–64. doi: 10.1084/jem.20111941 PMC3280879 22249450

[50] He J-S Meyer-Hermann M Xiangying D Zuan LY Jones LA Ramakrishna L. The distinctive germinal center phase of IgE+ b lymphocytes limits their contribution to the classical memory response. J Exp Med (2013) 210:2755–71. doi: 10.1084/jem.20131539 PMC3832920 24218137

[51] Hoof I Schulten V Layhadi JA Stranzl T Christensen LH Herrera de la Mata S. Allergen-specific IgG+ memory b cells are temporally linked to IgE memory responses. J Allergy Clin Immunol (2020) 146:180–91. doi: 10.1016/j.jaci.2019.11.046 PMC7860973 31883847

[52] Vijay R Guthmiller JJ Sturtz AJ Surette FA Rogers KJ Sompallae RR. Infection-induced plasmablasts are a nutrient sink that impairs humoral immunity to malaria. Nat Immunol (2020) 21:790–801. doi: 10.1038/s41590-020-0678-5 32424361 PMC7316608

[53] Glaros V Rauschmeier R Artemov AV Reinhardt A Ols S Emmanouilidi A. Limited access to antigen drives generation of early b cell memory while restraining the plasmablast response. Immunity (2021) 54:2005–2023.e10. doi: 10.1016/j.immuni.2021.08.017 34525339 PMC7612941

[54] Manz RA Thiel A Radbruch A. Lifetime of plasma cells in the bone marrow. Nature (1997) 388:133–4. doi: 10.1038/40540 9217150

[55] Kabashima K Haynes NM Xu Y Nutt SL Allende ML Proia RL. Plasma cell S1P1 expression determines secondary lymphoid organ retention versus bone marrow tropism. J Exp Med (2006) 203:2683–90. doi: 10.1084/jem.20061289 PMC2118149 17101733

[56] Gevaert P Nouri-Aria KT Wu H Harper CE Takhar P Fear DJ. Local receptor revision and class switching to IgE in chronic rhinosinusitis with nasal polyps. Allergy (2013) 68:55–63. doi: 10.1111/all.12054 23157682

[57] Testera-Montes A Palomares F Jurado-Escobar R Fernandez-Santamaria R Ariza A Verge J. Sequential class switch recombination to IgE and allergen-induced accumulation of IgE+ plasmablasts occur in the nasal mucosa of local allergic rhinitis patients. Allergy (2022) 77:2712–2724. doi: 10.1111/all.15292 35340036

[58] Fink K. Origin and function of circulating plasmablasts during acute viral infections. Front Immunol (2012) 3:78. doi: 10.3389/fimmu.2012.00078 22566959 PMC3341968

[59] Jacobi AM Mei H Hoyer BF Mumtaz IM Thiele K Radbruch A. HLA-DRhigh/CD27high plasmablasts indicate active disease in patients with systemic lupus erythematosus. Ann Rheum Dis (2010) 69:305–8. doi: 10.1136/ard.2008.096495 19196727

