# Supplementary material for: IgE+ plasmablasts predict the onset of clinical allergy
Source: Front Immunol. 2023 Feb 2;14:1104609. doi: 10.3389/fimmu.2023.1104609 (PMC9932261; doi:10.3389/fimmu.2023.1104609)
Supplement: Supplementary file 1 [file DataSheet_1.docx]

Supplementary Material

IgE+ plasmablasts predict the onset of clinical allergy

**Elisabeth M Simonin^1†^, Susanna Babasyan^1†^, Justine Tarsillo^1^, Bettina Wagner^1*^**

^1^Department of Population Medicine and Diagnostic Sciences, College of Veterinary Medicine, Cornell University, Ithaca, NY, USA

**^†^**These authors have contributed equally to this work and share first authorship.

**^*^Correspondence:**Bettina Wagner
bw73@cornell.edu

**
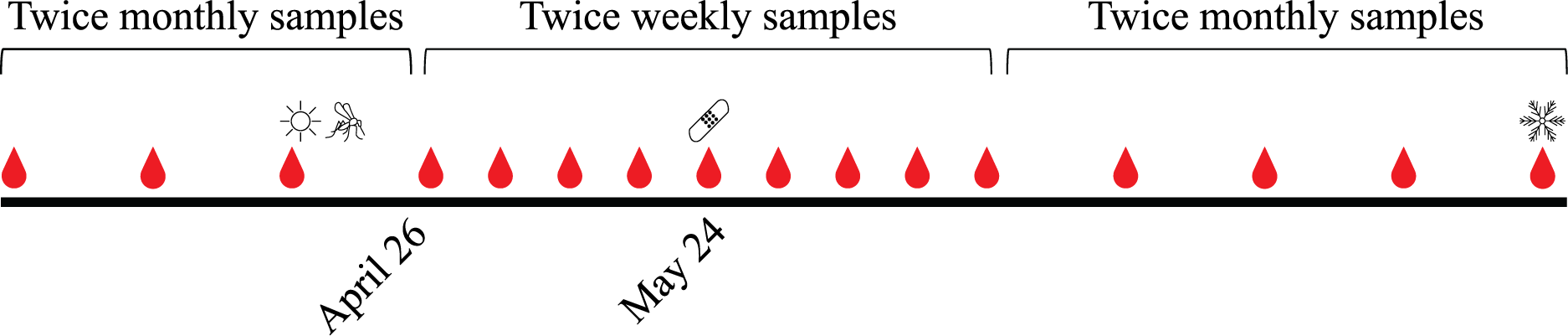
**

**Supplemental Figure 1: Timepoints of sample collection**

Samples were collected every 3-4 days from April 12 – June 10 when midges were in the environment. During this time, horses looked first clinically unaffected and then developed clinical allergy on or shortly after May 24. Before and after this period, samples were collected every 14 days until the first frost on November 2. Samples were collected once a month in February and December.
